# Zebrafish: A Cost-Effective Model for Enhanced Forensic Toxicology Capabilities in Low- and Middle-Income Countries

**DOI:** 10.7759/cureus.76223

**Published:** 2024-12-22

**Authors:** Sourik Mukherjee, Aman K Mohanty, Raj Kumar Chinnadurai, Dipayan Deb Barman, Abhijit Poddar

**Affiliations:** 1 Zebrafish Research Unit, Mahatma Gandhi Medical Advanced Research Institute, Sri Balaji Vidyapeeth (Deemed-to-be-University), Pondicherry, IND; 2 Forensic Medicine and Toxicology, Shri Sathya Sai Medical College and Research Institute, Sri Balaji Vidyapeeth (Deemed-to-be-University), Pondicherry, IND

**Keywords:** animal models, challenges, cost-effective, forensic toxicology, genetic similarity, high-throughput screening, low and middle-income countries (lmics), medical forensics, novel psychoactive substances (nps), zebrafish

## Abstract

Low- and middle-income countries (LMICs) are increasingly challenged by the rising burden of medicolegal cases. Traditional forensic infrastructure and in vivo rodent models often have significant limitations due to high costs and ethical concerns. As a result, zebrafish (*Danio rerio*) are gaining popularity as an attractive alternative model for LMICs because of their cost-effectiveness and practical advantages. Zebrafish have a lower acquisition cost, require less demanding husbandry, and have rapid development cycles, all of which facilitate faster and more economical toxicological studies, even in limited laboratory space. Additionally, the optical transparency of zebrafish embryos and larvae allows for non-invasive in vivo observations, reducing the need for extra resources. Research has shown that zebrafish can effectively investigate the behavioral, developmental, and cardiotoxic effects of various novel psychoactive substances (NPSs), including synthetic opioids, cathinones, and hallucinogens. They also excel in metabolic profiling, producing a broader range of metabolites than other models, with significant overlap in human metabolism. The presence of mammalian-like metabolic enzymes further positions zebrafish as a valuable tool for understanding human NPS metabolism and predicting potential effects. Notably, they can identify metabolites that traditional models may not detect, underscoring their potential for novel metabolite discovery. Despite these advantages, standardizing data collection protocols and addressing interlaboratory variability are crucial challenges that must be overcome for the widespread adoption of the zebrafish model. However, ongoing global efforts are paving the way to address these limitations and ensure the successful integration of zebrafish models into the field of forensic toxicology. This review highlights the potential of zebrafish as a cost-effective and versatile model for LMICs, emphasizing their growing application in NPS research and forecasting broader adoption in forensic toxicology.

## Introduction and background

Forensic toxicology is a multidisciplinary field that applies the principles and methods of toxicology, analytical chemistry, pharmacology, and clinical chemistry to help legal investigations [1]. It links scientific analysis with legal proceedings while analyzing toxins, drugs, and other foreign substances to determine their presence, concentrations, and impact at cellular and physiological levels [2]. Such information can be helpful in determining the cause of death, the type of drug used in an intoxication case, or the level of impairment in a driving under the influence investigation, which have a critical influence on criminal investigations and better public health outcomes [2,3].

The global rise of novel psychoactive substances (NPSs), often referred to as "emerging drug threats," presents a continuous challenge for forensic toxicologists [4]. NPSs are a constantly evolving class of synthetic substances designed to mimic or surpass the effects of traditional illicit drugs by altering perception, consciousness, mood, behavior, or cognition. Therefore, it necessitates developing new identification and detection methods to prevent them from going undetected. Moreover, over the past decade, forensic toxicology laboratories have witnessed a surge in demand for toxicological analyses, outpacing the allocation of resources [5]. This disparity between increasing demand and stagnant resources leads to inevitable consequences, including extended turnaround times and growing backlogs. However, global efforts to expand forensic laboratory capacity and secure additional funding primarily focus on resource-rich nations, leaving low- and middle-income countries (LMICs) with limited options [6].

The model organisms have been recognized as valuable tools, particularly in investigating alterations in post-mortem tissues in forensic toxicology [7]. A comprehensive analysis of forensic research conducted between 2012 and 2016 revealed the utilization of over 5,000 animals as human tissue analogs across 204 studies. Of these, rats (35.3%), pigs (29.3%), mice (17.7%), and rabbits (8.2%) emerged as the most frequently employed species, with the preferred choice often exhibiting a correlation with the specific research theme pursued [8]. However, traditional rodent-based toxicology testing presents significant challenges, particularly for LMICs. These challenges include substantial financial investments toward establishing, operating, and maintaining specialized animal facilities, animal care, and waste management. The acquisition of advanced equipment, such as individually ventilated cages, biosafety cabinets, telemetry systems, imaging systems like micro-CT scanners, MRI, behavioral testing equipment, mass spectrometers, ELISA readers, and flow cytometers often surpass the financial capabilities of LMICs [9]. Moreover, building trained human capacity for rodent handling and laboratory management is essential but resource-intensive. These limitations can hinder research and development, delay or impede legal investigations, and compromise public health by restricting the scope of toxicological analysis.

**Figure 1:**
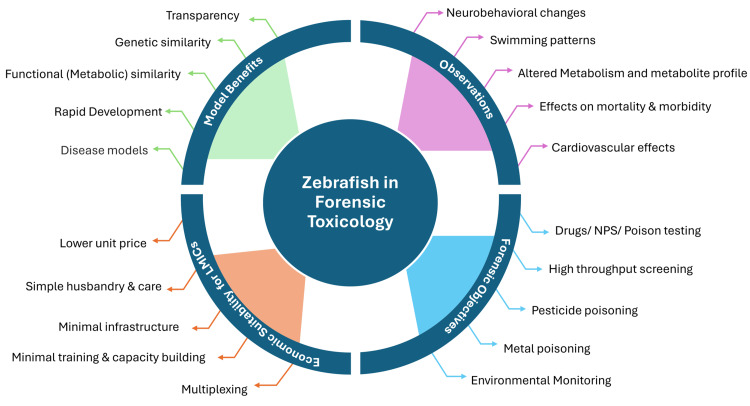
Transformative Role of Zebrafish in Forensic Toxicology (Prepared in This Study) NPS: novel psychoactive substance.

Therefore, enhancing forensic toxicology capabilities in LMICs necessitates a multifaceted approach. While long-term solutions involve increased funding, policy reforms, and capacity building through collaboration, a more immediate and sustainable strategy lies in exploring resource-efficient alternative models for toxicological analysis. In this regard, Zebrafish (*Danio rerio*) presents a compelling alternative, offering a cost-effective and accessible option compared to traditional rodent-based testing (Figure 1).

Today, zebrafish have become a cornerstone of modern biological research. Its unique biological features and amenability to genetic manipulation make it a valuable tool for understanding fundamental biological processes, modeling human diseases, and developing new drugs and therapies. Extensive research has been done in areas of developmental biology, gene functions, disease modeling, routine toxicology, and drug discovery processes [10-12].

## Review

About zebrafish

The zebrafish (*Danio rerio*) is a small freshwater fish native to South Asia, particularly abundant in India [13]. It belongs to the Cyprinidae family, a taxonomically diverse group that also encompasses commercially important aquaculture species like carp and rohu and popular ornamental fish such as the goldfish (*Carassius auratus*) [14]. In their natural habitat, zebrafish primarily occupy shallow, slow-moving to stagnant freshwater environments [15]. These include streams, canals, ditches, oxbow lakes, ponds, and even paddy fields. The water conditions they favor are near-neutral to slightly basic, with temperatures ranging from a comfortable 16.5°C to a balmy 34°C (61.7°F-93.2°F) [16].

As opportunistic omnivores, zebrafish exhibit a flexible diet [17]. Their primary food sources include zooplankton, phytoplankton, insects, and insect larvae. They readily adapt to seasonal variations in food availability by consuming worms and small crustaceans when preferred options are scarce. It offers flexibility in its ecological niche. Zebrafish play a crucial role in maintaining ecological balance within the aquatic ecosystem. They serve as prey for larger aquatic organisms while simultaneously contributing to population control of mosquito larvae and other small invertebrates through consumption [18].

**Figure 2:**
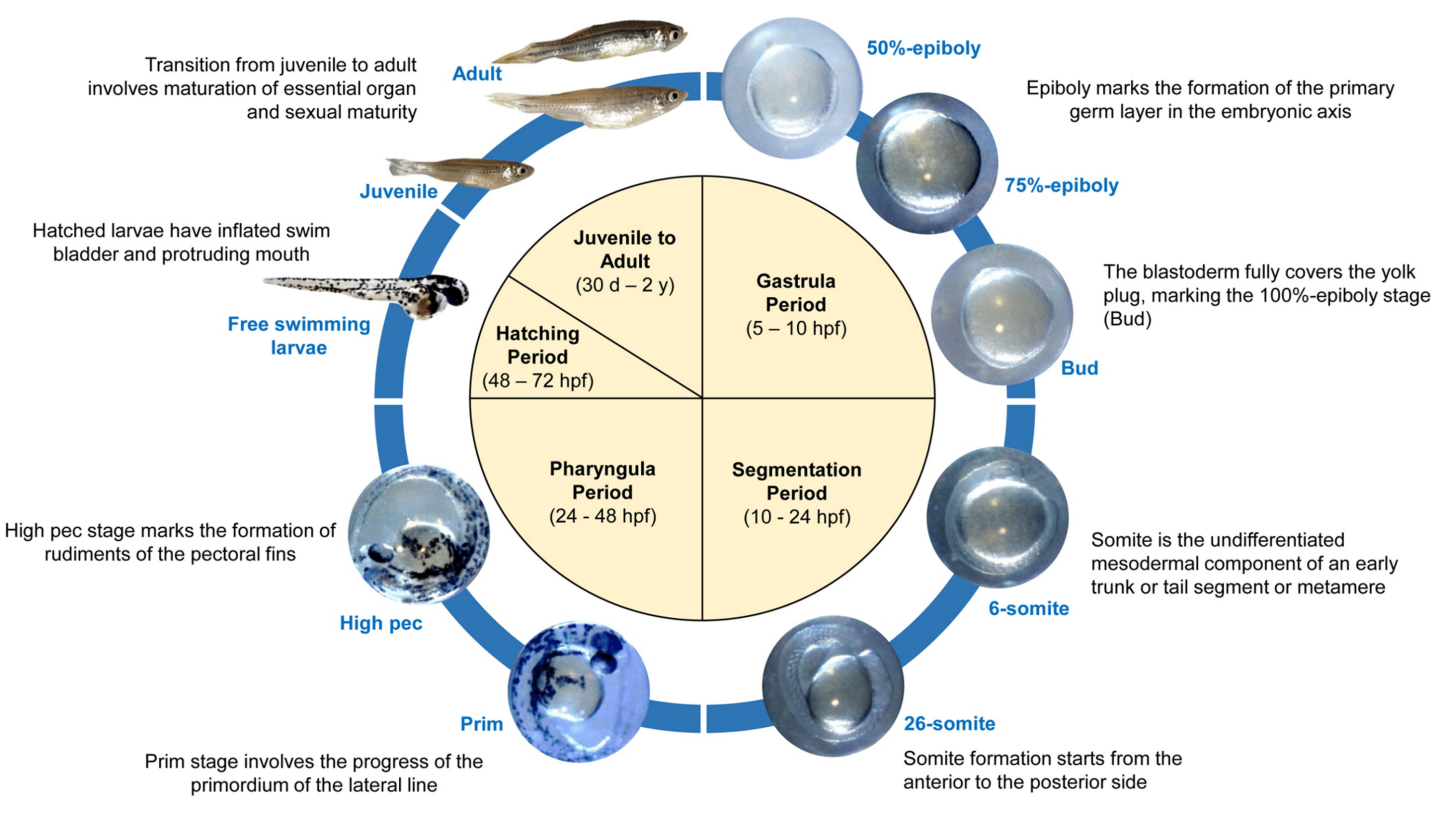
Zebrafish Life Cycle (Prepared in This Study) All images were taken at the Zebrafish Research Laboratory at our institution. Different developmental stages were mentioned in blue color. Highlights of each stage are mentioned in the figure. hpf, hours post fertilization.

Zebrafish are schooling fish, meaning that they live in social groups for safety and foraging efficiency [19]. These groups can be quite large, offering protection from predators. Within the shoal is a social hierarchy, with dominant individuals leading the group movements. Zebrafish are prolific breeders, spawning throughout the year in warmer climates. The zebrafish life cycle exemplifies a remarkable feat of rapid development (Figure 2). Females scatter hundreds of eggs in vegetation, and males fertilize them externally. Following fertilization, the embryo develops through well-defined stages within days. Initially, a single cell (zygote) progresses through cleavage, blastulation, and gastrulation, forming a basic body plan with a beating heart by day 2. Hatching as a free-swimming larva at day 3, the organism initially relies on its yolk sac for nutrition before developing a mouth and gills for feeding [20]. The larval stage witnesses continuous growth and organ development from the day of hatching and eventually transforms into a juvenile around day 30. Juveniles further mature, acquiring adult features and reproductive capability by day 60 [21]. Finally, at approximately three months old, the zebrafish reaches sexual maturity, becoming a fully functional adult with a potential lifespan of up to five years. This rapid life cycle and the transparency of embryos make zebrafish an ideal model organism for researchers to study developmental processes and the effects of various perturbations in real time [20].

For the scientific community, zebrafish first captured attention in the 1930s for their striped pigment patterns, which later offered insights into genetics and physical and cellular mechanisms [22]. These stripes, typically blue or gold on a silvery background, resemble the black and white stripes of a zebra (*Equus quagga*). While not an exact replication, the striped pattern warrants the comparison, solidifying the association within the name. However, the actual turning point for the zebrafish arrived in the 1960s with the pioneering work of George Streisinger [23]. Streisinger recognized the zebrafish's vast potential beyond its attractive pigmentation. His works laid the foundation for increased zebrafish research during the 1970s and 1980s. This growth was further amplified by the remarkable finding that zebrafish share approximately 70% of their genes with humans [24]. It opened doors for using zebrafish as a powerful model organism to understand human development and disease. By the late 1990s, the zebrafish's unique combination of rapid development, transparent embryos, and shared genetic heritage with humans solidified its position as a well-established model organism. Furthermore, the advent of powerful genetic tools like CRISPR-Cas9 enhanced the value of zebrafish, allowing scientists to manipulate genes and study their effects in real time, accelerating the pace of discovery [25].

Advantages of using zebrafish in forensic toxicology

Timely testing and reporting are critical for forensic toxicology. The passage of time can significantly degrade the integrity of biological samples, hindering the identification and quantification of substances relevant to an investigation. This temporal challenge necessitates rapid and efficient analysis techniques to preserve the evidentiary value of samples, preferably in models that have rapid development. In this direction, zebrafish hold the benefits over other rodent models. Unlike rodents, zebrafish embryos develop rapidly, reaching maturity in days. This swift lifecycle enables rapid assessment of toxin effects at various developmental stages, providing valuable insights into a toxin's mechanism of action [26]. It results in time benefits for the study even when biological samples from the victim are limited or degraded [27].

In forensic toxicology, the ability to observe and document toxin-induced changes in tissues and organs is crucial for accurate analysis. Traditional methods often rely on staining techniques or complex visualization procedures, which can be time-consuming and increase the overall cost of testing. Zebrafish present a significant advantage in this regard due to the remarkable transparency of their embryos and larvae [28]. This inherent transparency allows researchers to directly observe internal organs, tissues, and even cellular processes in real time using simple light microscopy. This eliminates the need for intricate dissection or tissue staining protocols that are commonly employed for studying opaque organisms. Furthermore, the transparency of zebrafish facilitates the direct visualization of how toxins impact internal structures. It gives insights into the mechanisms of action and early signs of toxicity [29]. This visual clarity surpasses traditional methods, offering a real-time window into the physiological impact of toxins on the living organism.

The degree of genomic homology with humans is crucial in selecting an appropriate animal model for forensic toxicology. This similarity allows for observations and outcomes obtained from the model to be extrapolated to human scenarios with greater confidence [30]. Zebrafish hold immense value in this regard, sharing approximately 70% of their genes with humans [24]. It indicates that a significant portion of their genes have been preserved throughout evolution. These conserved genes often play essential roles in fundamental biological processes, including metabolism, organ function, and cellular signaling pathways [24]. This remarkable genetic homology translates to similar toxicological responses, rendering zebrafish a reliable model for studying the effects of toxins on human health [31].

While approximately 70% shared gene pool between zebrafish and humans represents a compelling foundation for their use in toxicological studies, additional factors contribute to their suitability as models. The first of these factors lies in functional similarity. Shared genes often exhibit analogous functions at the cellular and physiological levels, extending beyond mere sequence homology [32]. This conservation of function strengthens the predictive power of zebrafish models in human toxicology, allowing for more reliable inferences about potential human responses to toxins [33].

Forensic toxicology is critical in legal investigations, as it analyses biological samples to identify and quantify toxins involved in poisoning cases. However, the presence of a toxin alone does not always paint the complete picture. Understanding the complex interactions between toxins and pre-existing medical conditions is crucial for accurately interpreting toxicological findings. Pre-existing conditions can alter toxins' absorption, distribution, metabolism, and excretion [34]. For example, liver disease can affect toxin metabolism, leading to increased blood concentrations and potentially more severe effects [35]. Likewise, certain conditions can exacerbate the toxic effects of a particular substance. For instance, heart disease may increase vulnerability to cardiac toxins, leading to more serious complications [36]. Moreover, pre-existing conditions can sometimes mimic the symptoms caused by a poison, making it difficult to distinguish between the two. To address these challenges, toxicologists searched for suitable disease models that mimic human conditions. In this, the shared genetic and functional landscape between zebrafish and humans largely facilitates the development of disease phenotypes in zebrafish that mimic various human pathologies [37]. These disease models offer a valuable tool for toxicologists to investigate toxins' impact on specific disease states, thereby creating a better legal decision-making process [8].

Potential of zebrafish for NPS investigations

Till November 2023, over 1,200 NPSs have been reported to the United Nations Office on Drugs and Crime (UNODC) Early Warning Advisory (EWA) [38]. This highlights the rapid emergence of NPS, with an estimated 950 NPSs released illegally onto the market in 2020, according to the World Drug Report 2021. While rodents remain a mainstay for evaluating NPS, zebrafish are gaining traction as a complementary model. Research in this field has demonstrated this paradigm shift, yielding substantial knowledge regarding drug-induced morphological and developmental abnormalities, behavioral alterations, cardiotoxicity, and metabolic alterations in embryonic, larval, and adult life stages [39,40].

Studies on Zebrafish Embryos

Traditionally, zebrafish embryos offer a distinct advantage over other vertebrate models due to their technical, economic, and ethical benefits [28]. Embryos bridge the gap between in vitro and in vivo studies by occupying a unique position in the regulatory landscape. Their early developmental stage, prior to three hours post-fertilization, places them outside the scope of cell culture regulations [41]. Conversely, their independence from external feeding post-hatching until day 5 exempts them from animal welfare regulations [42]. This enables embryos to serve as versatile platforms for investigating biological processes.

By leveraging these advantages, zebrafish embryos have increasingly been utilized in forensic toxicology to screen for the impacts of NPS. A study examining the toxicity of a phenethylamine (metaphedrone, 3-MMC) in zebrafish embryos found no mortality at concentrations ranging from 0.1 to 100 µg/L over 96 hours [43]. This contrasts with the dose-dependent mortality observed with fentanyl, and derivatives of phenethylamine (2-(4-chloro-2,5-dimethoxyphenyl)-N-[(2-methoxyphenyl)methyl]ethanamine (25C-NBOMe), 2,5-dimethoxy-N-((2-methoxyphenyl)methyl)benzeneethanamine (25H-NBOMe), and 2-({[2-(2,5-Dimethoxyphenyl)ethyl]amino}methyl)phenol (25H-NBOH)) [44-46]. Dose-dependent mortality was reported in zebrafish embryos, with a maximum mortality rate of 67% observed at a concentration of 18 μg/ml. Likewise, in *Artemia salina*, the highest mortality reached 82% at a concentration of 30 μg/ml [45].

Additional studies have determined 50% lethal concentration (LC_50_) values for various NPSs in zebrafish embryos. For example, the LC_50_ values for a synthetic cathinone (N-ethylpentylone (NEP)), and two opioids namely etazene (ETZ) and Morphine (MORPH) were reported to be 2.77 mM, 182.8 µM, and 25.21 mM, respectively [47,48]. In a similar study on 25C-NBOMe, the LC_50 _value was reported to be 10.76 μg/ml [45]. These studies also revealed morphological and developmental abnormalities, including delayed hatching, heart malformations, edema, craniofacial malformations, yolk edema, abnormal body shape, loss of pigmentation, and the development of a cylindrical heart [48]. Similarly, exposure to a synthetic cannabinoid (N-(adamantan-1-yl)-1-(5-fluoropentyl)-1H-indazole-3-carboxamide (5F-APINAC)) resulted in a lower hatching rate and malformations of the tail, spine, and hyperpigmentation [49]. The teratogenic potential of 25C-NBOMe has also been demonstrated, with lower, non-lethal concentrations of 25H-NBOMe and 25H-NBOH producing similar effects such as delayed hatching, blood clotting, and body malformations [45].

Furthermore, 3-MMC and fentanyl induce developmental defects in zebrafish embryos, including changes in eye area, tail abnormalities, yolk defects, and pericardium abnormalities [43,46]. The administration of 25C-NBOMe has also been linked to motor neuron abnormalities, highlighting the importance of carefully selecting appropriate doses in zebrafish larvae research on NPS [45]. A summary of the study findings is presented in Table 1.

**Table 1:** Forensic Toxicological Analysis of NPS in Zebrafish Embryos +: positive effects reported; -: no effects reported; NR, not reported; LC50: lethal concentration fatal for 50% of fish in a treatment group; 25H-NBOMe: 2,5-dimethoxy-N-((2-methoxyphenyl)methyl)benzeneethanamine; 25H-NBOH: 2-({[2-(2,5-dimethoxyphenyl)ethyl]amino}methyl)phenol; 25C-NBOMe: 2-(4-chloro-2,5-dimethoxyphenyl)-N-[(2-methoxyphenyl)methyl]ethanamine; NEP: N-ethylpentylone; 5F-APINAC: N-(adamantan-1-yl)-1-(5-fluoropentyl)-1H-indazole-3-carboxamide. Mortality includes embryo coagulation and formation of milky white embryos; malformation includes spine, pericardium, craniofacial, and tail deformities.

Novel Psychoactive Substance (NPS)	Mortality	Morphological and Developmental Abnormalities								LC_50_ value	References
		Edema	Yolk deformation	Irregular body curvature	Irregular heart rate	Irregular pigmentation	Delayed hatching	Malformation	Blood clot		
Metaphedrone	-	NR	NR	NR	NR	NR	NR	NR	NR	NR	[43]
25H-NBOMe	+	+	-	-	-	-	+	+	+	NR	[44]
25H-NBOH	+	-	-	-	-	-	+	+	-	NR	[44]
25C-NBOMe	+	+	+	+	+	+	-	-	-	10.76 μg/ml	[45]
Fentanyl	+	-	-	-	-	-	-	+	-	NR	[46]
NEP	+	NR	NR	NR	NR	NR	NR	NR	NR	2.77 mM	[47]
Etazene	+	+	-	-	+	+	-	+	-	182.8 μM	[48]
Morphine	+	-	-	-	-	-	-	-	-	25.21 mM	[48]
5F-APINAC	-	-	-	-	-	+	+	+	-	NR	[49]

Studies on Larvae

Studies conducted on zebrafish larvae have revealed varying effects of NPS on mortality, morphology, and development. While many of these substances do not induce significant mortality, they can cause a range of developmental abnormalities, cardiovascular dysfunction, and neurological impairments. Initial investigations indicated some drugs, including synthetic opioids (1-[4-[(E)-3-phenylprop-2-enyl]piperazin-1-yl]butan-1-one (AP-237), 2-methyl AP-237, isotonitazene, flunitazene, etodesnitazene, metonitazene, metodesnitazene, N-pyrrolidino etonitazene, butonitazene), synthetic cannabinoids (5F-APINAC and methyl (2S)-2-[[1-(4-fluorobutyl)indazole-3-carbonyl]amino]-3,3-dimethylbutanoate (4F-MDMB-BINACA)), demonstrated negligible to no mortality [49-52]. In contrast, drugs, including synthetic opioids, NEP, and 2-furanylfentanyl, induced developmental abnormalities like pericardial edema, yolk sac edema, somite and body axis deformities, spinal deformities, and swollen abdomens [47,52,53]. In a study, Park et al. [51] found that the administration of 4F-MDMB-BINACA leads to reduced liver size. Contrary to that Wagmann et al. [50] reported no morphological abnormalities associated with the administration of 4F-MDMB-BINACA. Likewise, the administration of ocfentanil did not lead to any morphological changes in the larvae [53]. All these studies showed that these drugs have different dose-dependent effects on mortality, morphology, or the development of the zebrafish larvae.

Furthermore, first-generation cathinones such as mephedrone, methylone, and methylenedioxypyrovalerone (MDPV), along with classical stimulants like cocaine (COC) and 3,4-methyl​enedioxy​methamphetamine (MDMA) drugs were found to induce bradycardia, arrhythmia, atrioventricular (AV) block, and signs of QT interval prolongation in larvae. Among them, MDPV exhibited a distinct pattern of rhythmicity changes and was the most potent in causing bradycardia and AV block compared to the other drugs [54]. Cardiotoxic effects, including arrhythmia, were also observed with the synthetic opioid ETZ and other drugs such as ephedrine (EPH) and COC [48,55]. NEP also triggered arrhythmia and exhibited dose-dependent cardiotoxicity, accompanied by decreased expression of *nkx2.5*, *nppa*, and *nppb*, which are associated with precardiac mesoderm markers, cardiac muscle cell proliferation, and cardiac stress, respectively. Additionally, NEP caused neurotoxicity, leading to a reduced neuron count and downregulation of *neurog1* genes [47]. EPH and COC are also reported to have negative impacts on the nervous system [55]. Likewise, NEP exposure in embryos downregulated genes crucial for brain development and caused anxiety-like behavior in zebrafish larvae [47]. COC and EPH exposure also impacted zebrafish behavior and neurotransmitter levels [55]. The neurotransmitter level alterations were also reported upon administration of 5F-APINAC in larvae [49]. This suggests the potential of zebrafish larvae for investigating the central nervous system (CNS) effects of novel synthetic opioids.

Zebrafish larvae also demonstrated promise in replicating behavioral effects observed in rodents. Fentanyl, APINAC, and methiopropamine (MPA) caused similar behavioral changes in both zebrafish and rodents [56,57]. Importantly, MPA and APINAC impaired spontaneous motor and sensorimotor behavior in both species [56]. Similarly, administration of 3-MMC, 2-furanylfentanyl, and ocfentanil resulted in locomotor behavioral changes indicative of anxiety [43,53]. 

In summary, the existing studies on NPS indicate contrasting effects on zebrafish larvae (Table 2). This warrants further research to fully understand the long-term consequences of NPS exposure in zebrafish larvae and to inform public health interventions.

**Table 2:** Forensic Toxicological Analysis of NPS in Zebrafish Larvae +: adverse effect; -: no effect; NR, not reported; CYPs: cytochrome P450s; GABA: gamma-aminobutyric acid; MDMA: 3,4-methyl​enedioxy​methamphetamine; MDPV: methylenedioxypyrovalerone; 4F-MDMB-BINACA: methyl (2S)-2-[[1-(4-fluorobutyl)indazole-3-carbonyl]amino]-3,3-dimethylbutanoate; AP-237: 1-[4-[(E)-3-phenylprop-2-enyl]piperazin-1-yl]butan-1-one; 5F-APINAC: N-(adamantan-1-yl)-1-(5-fluoropentyl)-1H-indazole-3-carboxamide. Mortality includes the total number of deaths; malformation includes deformities in liver, spine, brain, and heart and irregular jaw extension; behavioral alterations include hyperactivity and disorganized swimming patterns like speed changes, distance traveled, and absolute turn angle; cardiotoxicity includes bradycardia, arrhythmia, atrial-ventricular block, and signs of QT interval prolongation; and metabolic alterations mean changes in the level of metabolites compared to control.

Novel Psychoactive Substance (NPS)	Mortality	Morphological and Developmental Abnormalities				Behavioral Alterations	Cardiotoxicity	Metabolic Alterations	References
		Edema	Deformity	Malformation	Irregular pigmentation				
Metaphedrone	-	+	+	-	-	+	NR	NR	[43]
NEP	-	+	-	+	-	+	+	NR	[47]
Etazene	-	+	+	+	+	NR	+	CYPs	[48]
Morphine	-	+	-	+	-	NR	NR	NR	[48]
5F-APINAC	-	NR	NR	NR	NR	NR	NR	GABA, Glutamine, Tryptophan, Dopamine, Acetylcholine, Xanthurenic acid, Picolinic acid	[49]
4F-MDMB-BINACA	-	-	-	-	-	NR	NR	NR	[50]
4F-MDMB-BINACA	-	-	-	+	-	NR	NR	NR	[51]
AP-237	-	+	+	-	-	NR	NR	NR	[52]
2-methyl AP-237	-	+	+	-	-	NR	NR	NR	[52]
Isotonitazene	-	+	+	-	-	NR	NR	NR	[52]
Flunitazene	-	+	+	-	-	NR	NR	NR	[52]
Etodesnitazene	-	+	+	-	-	NR	NR	NR	[52]
Metonitazene	-	+	+	-	-	NR	NR	NR	[52]
Metodesnitazene	-	+	+	-	-	NR	NR	NR	[52]
N-pyrrolidino etonitazene	-	+	+	-	-	NR	NR	NR	[52]
Butonitazene	-	+	+	-	-	NR	NR	NR	[52]
Ocfentanil	-	-	-	-	-	+	NR	NR	[53]
2-furanylfentanyl	-	+	-	-	+	+	NR	NR	[53]
Cocaine	NR	NR	NR	NR	NR	NR	+	NR	[54]
MDMA	NR	NR	NR	NR	NR	NR	+	NR	[54]
Mephedrone	NR	NR	NR	NR	NR	NR	+	NR	[54]
Methylone	NR	NR	NR	NR	NR	NR	+	NR	[54]
MDPV	NR	NR	NR	NR	NR	NR	+	NR	[54]
Cocaine	NR	NR	NR	NR	NR	+	+	GABA, Dopamine	[55]
Ephedrine	NR	NR	NR	NR	NR	+	+	GABA, Dopamine	[55]

Studies on Adult Zebrafish

Compared to embryo and larval stages, the use of adult zebrafish in NPS research has been limited. To our knowledge, only three NPSs have been tested in adult zebrafish: a synthetic opioid (U-47700), a phenethylamine (Methamphetamine, Meth), and a cathinone (methylone) [58-60]. Studies investigating NPS-induced cardiotoxicity in adult zebrafish have demonstrated potential cardiac risks. For example, methamphetamine exposure increased heart rate, impaired heart function, and altered gene expression [58].

In an acute study, adult zebrafish exposed to increasing concentrations of U-47700 (1-50 mg/L) over 20 minutes exhibited a sedative effect. Conversely, chronic exposure (0.1-1 mg/L for 14 days) resulted in hyper-locomotion. Additionally, the study demonstrated U-47700's ability to cross the blood-brain barrier, with detectable drug levels found in brain samples after acute treatment with 1 and 10 mg/L [59].

Metabolite Investigation

Researchers are increasingly investigating the comparative metabolic profiles of NPS in different models to identify the most suitable system for understanding human metabolism and predicting potential effects. In this, zebrafish have emerged as a valuable model for studying NPS metabolism due to their ability to produce a wide range of metabolites, including both phase I and phase II metabolites. Numerous studies have employed zebrafish to analyze the metabolism of various NPS, including synthetic opioids (U-47700, valerylfentanyl, fentanyl), synthetic cannabinoids (methyl (2S)-2-[[1-(4-fluorobutyl)indole-3-carbonyl]amino]-3,3-dimethylbutanoate (4F-MDMB-BICA), 4F-MDMB-BINACA, methyl (2S)-2-[[1-(cyclohexylmethyl)indazole-3-carbonyl]amino]-3,3-dimethylbutanoate (MDMB-CHMINACA), (2S)-2-[[2-[1-[(4-fluorophenyl)methyl]indol-3-yl]acetyl]amino]-3,3-dimethylbutanamide (ADB-FUBIATA)), a derivative of lysergic acid (1cP-LSD), a compound belonging to tryptamine class (4-AcO-DET), cathinones (NEP, dipentylone), designer benzodiazepines (Clonazolam, Etizolam) and 3-Methoxyeticyclidine [46,48,51,59-71] (Table 3).

**Table 3:** Metabolite Investigation Using Zebrafish A: metabolites identified from liver; B: metabolites identified from brain; 4F-MDMB-BINACA: methyl (2S)-2-[[1-(4-fluorobutyl)indazole-3-carbonyl]amino]-3,3-dimethylbutanoate; α-PVP: α-pyrrolidinovalerophenone; 4-CDC: 4-chloro-N,N-dimethylcathinone;  AP-237: 1-[4-[(E)-3-phenylprop-2-enyl]piperazin-1-yl]butan-1-one; NEP: N-ethylpentylone; 4F-MDMB-BICA: methyl (2S)-2-[[1-(4-fluorobutyl)indole-3-carbonyl]amino]-3,3-dimethylbutanoate; ADB-FUBIATA: (2S)-2-[[2-[1-[(4-fluorophenyl)methyl]indol-3-yl]acetyl]amino]-3,3-dimethylbutanamide; 1cP-LSD: (6aR,9R)-4-(cyclopropanecarbonyl)-N,N-diethyl-7-methyl-6,6a,8,9-tetrahydroindolo[4,3-fg]quinoline-9-carboxamide; 4-AcO-DET: [3-[2-(diethylamino)ethyl]-1H-indol-4-yl] acetate; MDMB-CHMINACA: methyl (2S)-2-[[1-(cyclohexylmethyl)indazole-3-carbonyl]amino]-3,3-dimethylbutanoate.

Novel Psychoactive Substance (NPS)	Life Stage	Number of Metabolites Detected	References
Fentanyl	Larvae	3	[46]
Etazene	Larvae	1	[48]
4F-MDMB-BINACA	Larvae	18	[50]
4F-MDMB-BINACA	Larvae	18	[51]
AP-237	Larvae	1	[52]
2-methyl AP-237	Larvae	1	[52]
Isotonitazene	Larvae	2	[52]
Flunitazene	Larvae	1	[52]
Etodesnitazene	Larvae	1	[52]
Metonitazene	Larvae	1	[52]
Metodesnitazene	Larvae	2	[52]
N-pyrrolidino etonitazene	Larvae	2	[52]
Butonitazene	Larvae	4	[52]
Ocfentanil	Larvae	7	[53]
2-furanylfentanyl	Larvae	6	[53]
α-PVP	Adult	5	[60]
NEP	Adult	3	[60]
4-CDC	Adult	3	[60]
4F-MDMB-BICA	Larvae	18	[61]
Valerylfentanyl	Larvae	19	[62]
Clonazolam	Larvae	8	[63]
Etizolam	Adult	28	[64]
Dipentylone	Larvae	14	[65]
ADB-FUBIATA	Larvae	18	[66]
1cP-LSD	Larvae	3	[67]
4-AcO-DET	Larvae	12	[67]
Fentanyl	Adult	16A, 8B	[68]
3-Methoxyeticyclidine	Larvae	14	[69]
4F-MDMB-BINACA	Larvae	14	[70]
4F-MDMB-BICA	Larvae	16	[70]
MDMB-CHMINACA	Larvae	29	[71]

A study employed Meth to develop a method for analyzing an entire class of drugs. The author measured the internal concentration of Meth in zebrafish larvae using mixed-matched calibration, combined with extraction and purification through a quick and safe technique in liquid chromatography-tandem mass spectrometry. The study found that internal concentrations increased over time, with greater accumulation in the head compared to the trunk [72].

A key advantage of zebrafish larvae is their ability to produce a wide range of metabolites. A recent study investigated how different models, such as zebrafish larvae, human liver S9, HepaRG cells, processed substances like a compound hybrid of amphetamine and phenethylamine (amphetamine-3,4-DMA-NBOMe), a synthetic cathinone (ephylone), a synthetic cannabinoid (4F-MDMB-BINACA), a pyrrolidinophenone derivative (4F-PHP), and a lysergamide (1P-LSD). Among all, zebrafish larvae produced the most metabolites (around 79) encompassing both phase I (oxidation, N-demethylation, etc.) and phase II (sulfation, glucuronidation, etc.) processes. Notably, these metabolites showed the greatest overlap with those detected in human samples, including plasma and urine [50]. This suggests that zebrafish larvae can be valuable for comprehending human NPS metabolism.

A similar study using the Zebrafish Water Tank (ZWT) model was conducted to investigate the metabolism of xylazine. Samples were analyzed using liquid chromatography paired with high-resolution mass spectrometry. The findings revealed 11 phase I and II metabolites, four of which had been previously identified in humans. Additionally, abundant hydroxylated and oxygenated derivatives were detected, which could be targeted in doping controls in cases of misuse [73]. Stanozolol, a frequently misused androgenic anabolic steroid in sports, has been banned. The ZWT model is capable of detecting phase I metabolites. The ZWT model was utilized to identify phase II metabolites. The results revealed the highest concentrations of two phase II metabolites, which are glucuronide metabolite derived from the anabolic steroid stanozolol (17epi-STAN-N-Glucuronide, and STAN-O-Glucuronide) [74].

Zebrafish also possess metabolic enzyme systems with significant similarities to mammals. Enzymes like Cytochrome P450, crucial for initial drug metabolism in humans, and phase II enzymes, such as uridine glucuronosyltransferases (UGTs), sulfotransferases (SULTs), and methyltransferases (COMTs), are also present in zebrafish. Additionally, zebrafish's gene expression profiles of key phase II enzymes (UGTs, SULTs, COMTs) resemble their human counterparts [75-77]. This similarity allows zebrafish to perform phases I and II metabolism reactions, mimicking human processes.

Finally, several studies have shown that zebrafish can identify metabolites that are not detected in traditional in vitro models like human liver microsomes. For example, a study investigating the synthetic cannabinoid 4F-MDMB-BICA found a phase II metabolite present in zebrafish and human samples but absent in human liver microsomes [61]. This highlights the potential of zebrafish for discovering novel phase I and phase II metabolites of NPS.

Economic advantages

While the primary focus lies on the biological and medical aspects of forensic toxicology, it is essential to acknowledge the underlying economic considerations within a laboratory setting. In fact, this comes first when setting up and operating a laboratory in resource-constrained nations [78]. LMICs often have limited government budgets that call for balancing national development with ongoing challenges. Therefore, the laboratory must be sustainable by prioritizing lab functions and equipment [79]. Other factors like maintenance costs, staffing needs, and the ongoing cost of supplies also play a significant role. For example, the total recurring and non-recurring cost estimate for establishing a toxicology lab at All India Institute of Medical Sciences (AIIMS) Bhopal, India, was nearly a million dollar [80]. It is difficult for many small resource-constrained nations to afford such expenses. However, it is essential to scale up forensic toxicological laboratories to meet the increasing spectrum of toxic substances and the parallel rise of legally demanding tests. Therefore, cost-effectiveness in operations is of paramount significance, and precisely here, zebrafish find its suitability in comparison with currently used rodent models [81].

Zebrafish are relatively inexpensive compared to other models. For instance, our experience indicates a significant cost disparity between zebrafish and rodents in India. While zebrafish exhibit a significantly lower unit cost of $0.18 to $0.24, rodents command a price range of $2.38 to $7.14 per animal. Given the typical sample size of five to 10 rodents or 10-20 zebrafish per study, the overall cost reduction for zebrafish models can reach 90%-95% in terms of animal acquisition alone. Overall, the zebrafish model can reduce the cost of in vivo drug screening by nearly 500 times compared to rat models [82]. Zebrafish require minimal space and infrastructure and have simpler husbandry needs than traditional rodent models. The facility only needs a quarantine system, housing system, temperature monitoring, lighting, water quality control, and breeding equipment. Further, it is crucial to monitor the stocking density in each tank, ensure appropriate environmental enrichment, and maintain a regular feeding schedule [83]. In fact, the cost of building a zebrafish facility consisting of 27 tanks, each with a capacity of 1.8 liters, is approximately $1,500 [84]. Even, working with zebrafish requires less specialized training than working with rodents. As such, LMICs, with a limited budget for laboratory setting, operation, or upgradation, might struggle to establish a rodent toxicology facility yet can afford a functional zebrafish-based toxicology unit. The advantages add up given that rapid development and small and transparent sizes allow study completion in much less time and without sophisticated analytical equipment. For this, zebrafish assays can be performed with more samples than rodents [85]. This allows for faster and more cost-effective screening of potential toxins. It improves turnaround time while enabling a larger pool of sample testing. For example, a case of suspected pesticide poisoning in a rural community can be quickly addressed through zebrafish testing. It could accommodate a larger number of samples from different sources (water, food, victim's tissue) within a comparatively minimal budget, offering a more comprehensive analysis.

Future potential and gap identification

Zebrafish have emerged as a prominent model organism in contemporary toxicological research and industrial applications. This has led to their widespread use in acute toxicity testing, adhering to internationally accepted protocols established by the Organization for Economic Co-operation and Development (OECD). These protocols include the Testing of Chemicals with the Fish Acute Toxicity Test (OECD 203) and The Fish Embryo Acute Toxicity Test (OECD 236) [86]. Furthermore, zebrafish have demonstrated remarkable utility in high-throughput screening programs like the US Environmental Protection Agency's (US EPA) Toxicity Forecaster (ToxCast) initiative [87]. Zebrafish's efficiency in this program highlights their potential as robust biosensors, contributing valuable data to the public database of in vitro medium- and high-throughput screening assays. This data signified that zebrafish can prioritize and characterize the potential hazards of several chemicals and hold immense potential to transform forensic toxicology.

Within the field of forensic toxicology, the zebrafish has emerged as a valuable model organism for investigating the effects of NPS. With the ever-increasing global prevalence of NPS, zebrafish-based investigations are anticipated to play an increasingly prominent role in NPS research. Despite these advantages, zebrafish application in forensic settings remains in its early stages. This is primarily driven by the stringent data quality requirements of forensic toxicology laboratories. Test results from these labs hold immense legal weight, potentially influencing court decisions and impacting public safety [88]. Inaccurate data could lead to wrongful convictions or the release of criminals. Furthermore, toxicology results can be crucial for guiding medical decisions in poisoning or overdose cases. Robust data quality ensures healthcare professionals receive reliable information for effective treatment plans.

In such rigorous settings, zebrafish currently lack merit. Notably, there are considerable differences between zebrafish and humans in terms of disease development, drug response, and the applicability of findings to human outcomes [89]. Additionally, a broader range of differences can be seen in organ development, including sex differentiation, adaptive immunity, behavior, and neurology [90]. Thus, when studying complex mammalian systems, immune responses, and behavioral effects, the use of rodent models is crucial. Furthermore, unlike established rodent models, zebrafish studies lack standardized data collection and reporting practices. This interlaboratory variability leads to inconsistencies [91]. Different laboratories examine the same chemical and endpoint (biological effect) but report varying results due to internal lab standards. Recognizing this challenge, the Society for Environmental Applications of ZebraFish (SEAZIT) organized an information session on "Implementation of Zebrafish Ontologies for Toxicological Screening" in 2017. It revealed that, from a data science perspective, many seemingly disparate observations could be mapped to similar endpoints, with variations only in measurement timing, methodology, or terminology. With this insight, a web-based resource, the Zebrafish Phenotype Atlas, was proposed to harmonize data collection and represent endpoints and metadata [92]. Unfortunately, such a resource remains undeveloped, highlighting the ongoing need for standardized data collection and analysis in zebrafish toxicology, particularly for applications in forensic settings where robust and interoperable data is essential.

Encouragingly, ongoing research explores the potential of zebrafish in understanding the symptomatology and mechanisms of action associated with pesticide poisoning, suggesting broader applicability in toxicological analysis [93]. While this type of research is still underdeveloped in low- and middle-income countries (LMICs), upper-middle-income countries (UMICs) such as Brazil, Poland, and Colombia have extensively adopted zebrafish models for screening novel psychoactive substances (NPS) [45,48,60]. In Brazil, the Federal University of Rio de Janeiro partnered with the Civil Police of Rio de Janeiro State and the National Institute of Criminalistics of the Brazilian Federal Police to investigate the metabolism of synthetic cathinones using a zebrafish water tank model [60]. The Medical University of Lublin, in collaboration with the Institute of Toxicology Research in Borowa, the Department of Forensic Medicine at Wroclaw Medical University, and the Polish Police, carried out studies using zebrafish to investigate the toxic effects induced by ETZ [48]. These examples highlight the significance of zebrafish as a valuable alternative in forensic toxicology, particularly in LMICs, where cost-effective yet reliable methods are essential.

## Conclusions

Zebrafish have the potential to become a mainstay in toxicological analysis, not just for LMICs but for the entire field of forensic toxicology. Their efficiency, sensitivity, and adaptability position them as valuable tools for tackling the challenges of today and preparing for the complex forensic threats of tomorrow. However, overcoming current limitations will be crucial for their widespread adoption in forensic toxicology. Implementing standardized protocols and data quality control measures will ensure reliable and reproducible results across laboratories, fostering trust in the model for forensic applications. Furthermore, integrating cutting-edge technologies like high-throughput screening and advanced genetic analysis can significantly enhance efficiency and provide deeper insights into toxin-induced effects. It can even be used to predict the toxicological effects of newly identified compounds and develop more specific forensic assays for a broader range of toxins encountered in forensic casework.

The impact of zebrafish extends beyond LMICs, with the potential to revolutionize forensic toxicology as a whole. Faster turnaround times and increased sensitivity can lead to reduced case backlogs and improved detection of low-level toxins, expediting the justice process. Additionally, zebrafish models are not limited to traditional toxins. Their ability to mimic complex biological processes allows them to investigate the effects of emerging threats like NPS and environmental contaminants. This requires a multidisciplinary approach, fostering collaboration between toxicologists, biologists, and geneticists to unlock the full potential of zebrafish in complex forensic investigations. A global convergence of stakeholders will be essential for reaching a consensus and developing the roadmap for a better forensic toxicology regime, especially at LMICs.
